# Litchi–associated Acute Encephalitis in Children, Northern Vietnam, 2004–2009

**DOI:** 10.3201/eid1811.111761

**Published:** 2012-11

**Authors:** Juliette Paireau, Nguyen Hai Tuan, Rémi Lefrançois, Matthew R. Buckwalter, Ngu Duy Nghia, Nguyen Tran Hien, Olivier Lortholary, Sylvain Poirée, Jean-Claude Manuguerra, Antoine Gessain, Matthew L. Albert, Paul T. Brey, Phan Thi Nga, Arnaud Fontanet

**Affiliations:** Institut Pasteur, Paris, France (J. Paireau, R. Lefrançois, M.R. Buckwalter, J.-C. Manuguerra, A. Gessain, M.L. Albert, A. Fontanet);; National Institute of Hygiene and Epidemiology, Hanoi, Vietnam (N.H. Tuan, N.D. Nghia, P.T. Nga, N.T. Hien);; Université Paris X-Nanterre, Paris (R. Lefrançois);; INSERM U818, Paris (M.R. Buckwalter, M.L. Albert);; Hôpital Necker-Enfants Malades, Paris (O. Lortholary, S. Poirée);; Université Paris Descartes, Paris (O. Lortholary, M.L. Albert);; CNRS URA 1930, Paris (A. Gessain);; Institut Pasteur, Vientiane, Lao People’s Democratic Republic (P.T. Brey);; and Conservatoire National des Arts et Métiers, Paris (A. Fontanet)

**Keywords:** Encephalitis, epidemics, disease outbreaks, epidemiology, risk factors, litchi, retrospective studies, Vietnam, Asia, viruses, vector-borne infections

## Abstract

Since the end of the 1990s, unexplained outbreaks of acute encephalitis in children coinciding with litchi harvesting (May–July) have been documented in the Bac Giang Province in northern Vietnam. A retrospective ecologic analysis of data for 2004–2009 involving environmental, agronomic, and climatic factors was conducted to investigate the suspected association between the outbreaks and litchi harvesting. The clinical, biological, and immunologic characteristics of the patients suggested a viral etiology. The ecologic study revealed an independent association between litchi plantation surface proportion and acute encephalitis incidence: Incidence rate ratios were 1.52 (95% CI 0.90–2.57), 2.94 (95% CI 1.88–4.60), and 2.76 (95% CI 1.76–4.32) for second, third, and fourth quartiles, respectively, compared with the lowest quartile. This ecologic study confirmed the suspected association between incidence of acute encephalitis and litchi plantations and should be followed by other studies to identify the causative agent for this syndrome.

## Abstract 2

Outbreaks are spatiotemporally associated with litchi harvest, but the causative agent remains unknown.

Acute encephalitis syndrome (AES) is a major public health problem in Asia. The main etiologic agent is the Japanese encephalitis virus (JEV), a positive-sense single-stranded flavivirus transmitted by *Culex* spp. mosquitoes. It is responsible for ≈50,000 encephalitis cases every year in the region (*1*). Recently, the Nipah and Chandipura viruses were identified as responsible for acute encephalitis outbreaks in Malaysia and India (*2*,*3*). In addition, many other viral encephalitis cases of unknown etiology exist throughout Asia (*4*).

In Vietnam, according to the National Institute of Hygiene and Epidemiology (NIHE), the annual incidence rate for AES in the general population was 2.24–2.90 cases per 100 000 inhabitants during 1998–2005. This rate corresponds with 1,800–2,300 cases per year, two thirds of which occurred in northern Vietnam. Since the inclusion in 1997 of the JEV vaccine into the Extended Program on Immunization by the World Health Organization (WHO), the relative proportion of non-JE cases has increased substantially among patients hospitalized with AES in Vietnam, from ≈40% in 1996 to ≈90% in 2009 (P.T. Nga, unpub. data).

In northern Vietnam, unexplained outbreaks of non-JE acute encephalitis have been documented since 1999. These outbreaks are unusual because of their specific location (Bac Giang Province), their strict seasonality (92% of unexplained AES occur during May–July), the restricted age group of persons at risk (88% are <15 years old), and the clinical features (abrupt febrile onset, rapid progression to coma, and higher case-fatality rate than for JE). Approximately 50–100 children are referred to the provincial hospital each epidemic season, but the actual number of cases could be underreported because some patients might have died at home.

The local population and public health practitioners have anecdotally attributed the emergence of AES to the recent intensification of litchi production in the province: production rose from 870 tons during 1985–1989 to 400,000 tons during 2000–2005. Bac Giang Province has the highest litchi production in Vietnam, three fourths of which is consumed domestically and the rest is exported mainly to People’s Republic of China (*5*,*6*). Vietnamese litchis are mostly of the Thieu variety, which has a short harvest period of ≈1 month during May–July (*7*), which coincides with the epidemic season of the outbreaks in Bac Giang.

Because of the distinct early clinical manifestations (*8*), the syndrome has been locally termed Ac Mong encephalitis (AME), after the Vietnamese word for nightmare. The typical clinical illness starts with headache and fever, followed by seizures (often during the night); approximately one third of cases progress to coma and death.

The causative agent of AME has remained unidentified and may be responsible for unexplained acute encephalitis elsewhere in the world, particularly in regions sharing similar ecology and environment. Litchi is widely distributed throughout subtropical and tropical regions. The 5 leading litchi-producing countries are China, India, Taiwan, Thailand, and Vietnam (*9*).

Our first objective was to describe the epidemiologic and clinical features of this severe encephalitis among children in northern Vietnam. Our second objective was to strengthen or weaken the hypothesis that litchi cultivation is associated with acute outbreaks of AES in Bac Giang Province. We investigated this suspected association using a retrospective ecologic analysis for 2004–2009 in Bac Giang Province that involved various environmental, agronomic, and climatic factors. Confirmation of this association would pave the way for further hypothesis-testing studies investigating the causal mechanisms behind this ecologic correlation.

## Materials and Methods

### Study Area

**Figure 1:**
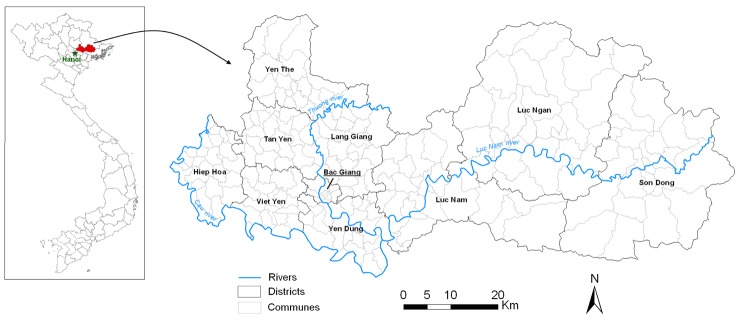
Location and map of Bac Giang province in Vietnam.

Bac Giang Province is located in northeastern Vietnam, 50 km from the capital, Hanoi (Figure 1). This mainly rural area covers 3,827 km^2^ and has a population of ≈1.6 million inhabitants. About one third of the land area is devoted to agriculture and one fourth to forestry and timber production. The climate comprises 2 distinct seasons: the cold, dry season from October through March and the hot, rainy season from April through September. The province is divided into 10 districts and 230 communes. The only hospital is in Bac Giang City, which is the capital of the province.

### Data Collection and Laboratory Analyses

Records from patients admitted to the provincial hospital with suspected AES from January 2004 through December 2009 were collected from Bac Giang Preventive Medicine Centre and NIHE. Among these patients, criteria for inclusion in the ecologic analyses were clinical diagnosis of suspected acute viral encephalitis, age <15 years, onset date during May 1–July 31, and negative serology for JEV IgM (IgM antibody-capture ELISA) or full immunization against JEV (receipt of 3 doses). Patients meeting these criteria are referred to as suspected AME case-patients.

Detailed clinical and biologic examinations, including blood analyses, cerebrospinal fluid (CSF) analyses, and cranial computed tomography scans, were available for 88 AME patients hospitalized during 2006–2009 that were included in a parallel case–control study (in progress). Alanine aminotransferase and aspartate aminotransferase levels also were tested in 17 patients. The representativeness of this subgroup was evaluated by comparing it with the whole group on all common measured characteristics (age, sex, day of onset, and geographic coordinates) by χ^2^ and *t* tests.

To determine the nature of the host response to AME, Multiple Analyte Profiling (Luminex Inc., Austin, TX, USA) was performed on the serum and CSF of a subset of AME patients collected in 2007. Samples were analyzed for 39 separate chemokines and cytokines by using the Luminex 100IS technology (Miliplex MAP kits; Milipore, Billerica, MA, USA). The analyzed group comprised samples from 5 patients who survived, 5 who died of the disease, and 4 serum samples from asymptomatic siblings. Data are presented as concentration (pg/mL). We used a Mann-Whitney U test to compare values between groups.

In virology, a collection of 9 supernatants from short-term cell cultures (including Vero and C6/36 cells) and their corresponding CSF and serum samples, taken from patients during 2006–2008 who had severe, including fatal, forms of disease, were all screened for known viruses by using PCR-based assays at Institut Pasteur (Paris, France). Searched viruses included JEV, dengue viruses 1–4, West Nile virus, chikungunya virus, herpes simplex viruses 1 and 2, varicella-zoster virus, Rift Valley fever virus, Crimean-Congo hemorrhagic fever virus, and most viruses of genera *Enterovirus* and *Alphavirus*. All assay results were negative.

### Ecologic Data Collection and Mapping

Demographic and agronomic data were collected by the Bac Giang Preventive Medicine Centre from the district statistics offices for each year from 2004 through 2009 for the 230 communes. These data included population count; production of rice and litchi; surface area dedicated to rice cultivation, litchi cultivation, and forestry; and the number of pigs, water buffaloes, cattle, and poultry. Starting and ending months of the rice and litchi harvests were collected at the district level. Daily meteorologic data were collected at the provincial level from the National Centre for Hydro-Meteorological Forecasting, Ministry of Natural Resources and Environment (Hanoi, Vietnam). These data included mean temperature, mean relative humidity, and total rainfall during January 2004–December 2009.

A commune-level map of Bac Giang Province was retrieved from the Map Library of the WHO health mapping program. To improve the accuracy of the WHO map, a 1:50,000 topographic map of Bac Giang Province (Cartography Office of Vietnam National Institute of Geography) was digitized and geo-referenced (WGS84 coordinate system) with SavGIS software (www.savgis.org). From these 2 cartographic sources, a new, more precise, map of Bac Giang communes and rivers (WGS84-UTM48N projection) was created with ArcGIS software (version 10.1, ESRI Inc., Redlands, CA, USA). In addition, mean elevations of the communes were derived from satellite raster data (US Geological Survey, Shuttle Radar Topography Mission), collected from the FTP server of Global Land Cover Facility, University of Maryland (ftp://ftp.glcf.umiacs.umd.edu/glcf/).

### Statistical Analyses

To investigate the spatiotemporal association between AME incidence and potential ecologic factors, including litchi cultivation, we conducted a commune-level retrospective ecologic regression analysis in Bac Giang Province for 2004–2009. A 2-level (year and commune) binomial negative regression model with fixed-effect coefficients and random-effect intercepts was conducted by using STATA software (version 11.0; StataCorp LP, College Station, TX, USA). The outcome was the number of AME cases per commune per year. The covariates included in the analysis are listed in Table 1. Continuous variables were modeled as categorical variables (by quartiles for agronomical variables and by terciles for meteorologic variables). Population size was introduced as an offset in the model so that the measure comparing the risk across exposure categories was an incidence rate ratio (IRR). The multivariate analysis included covariates with a p value <0.25 in the univariate analyses, and a backward deletion of variables by Wald test was conducted until all variables in the final model had p values <0.05.

**Table 1:** Univariate analyses of Ac Mong encephalitis, Bac Giang Province, Vietnam, 2004–2009

Variable/category	IRR (95% CI)*	p value†
Mean altitude, m		
<9.85	1	<0.001
9.85–16.47	1.23 (0.76–1.99)	
16.47–58.56	1.35 (0.83–2.20)	
>58.56	2.93 (1.88–4.56)	
Presence of a river in the commune		
No	1	0.996
Yes	1.00 (0.70–1.43)	
Litchi surface, % commune surface		
<1.49	1	<0.001
1.49–5.84	1.27 (0.75–2.15)	
5.84–12.92	2.89 (1.82–4.58)	
>12.92	2.73 (1.72–4.35)	
Rice surface, % commune surface		
<6.22	1	<0.001
6.22–24.51	0.64 (0.42–0.98)	
24.51–38.42	0.61 (0.40–0.93)	
>38.42	0.29 (0.17–0.48)	
Forest surface, % commune surface		
<0.48	1	<0.001
0.48–11.13	1.81 (1.06–3.09)	
11.13–40.58	3.32 (2.00–5.48)	
>40.58	4.05 (2.45–6.70)	
Litchi yield, tons/km^2^		
<157.68	1	0.083
157.68–300.02	1.18 (0.75–1.84)	
300.02–503.51	0.97 (0.61–1.54)	
>503.51	1.42 (0.93–2.17)	
Rice yield, tons/km^2^		
<460.00	1	0.500
460.00–500.00	0.83 (0.54–1.27)	
500.00–534.15	0.78 (0.50–1.21)	
>534.15	0.68 (0.43–1.08)	
Pigs density, no./km^2^		
<178.11	1	
178.11–419.53	0.45 (0.29–0.69)	<0.001
419.53–681.32	0.47 (0.31–0.71)	
>681.32	0.29 (0.18–0.47)	
Poultry density, no./km^2^		
<1,449.79	1	<0.001
1,449.79–3,715.18	0.57 (0.38–0.85)	
3,715.18–7,065.25	0.58 (0.39–0.86)	
>7,065.25	0.21 (0.12–0.37)	
Buffaloes density, no./km^2^		
<11.57	1	0.050
11.57–22.85	1.91 (1.18–3.11)	
22.85–35.06	1.82 (1.10–2.98)	
>35.06	1.72 (1.04–2.84)	
Cattle density, no./km^2^		
<7.04	1	<0.001
7.04–49.66	0.78 (0.53–1.14)	
49.66–105.12	0.43 (0.28–0.67)	
>105.12	0.27 (0.16–0.44)	
Mean temperature, °C		
January–April		
<19.23	1	0.004
19.23–20.16	0.74 (0.53–1.04)	
>20.16	0.53 (0.37–0.77)	
May–August		
<27.88	1	<0.001
27.88–28.53	0.32 (0.21–0.49)	
>28.53	0.81 (0.59–1.12)	
Mean humidity, %		
January–April		
<82.07	1	<0.001
82.07–83.55	0.81 (0.59–1.12)	
>83.55	0.32 (0.21–0.49)	
May–August		
<82.96	1	0.059
82.96–83.84	0.72 (0.51–1.02)	
>83.84	0.68 (0.47–0.97)	
Total rainfall, mm		
January–April		
<150	1	0.059
150–210	0.94 (0.64–1.37)	
>210	1.38 (0.98–1.96)	
May–August		
<518	1	0.059
518–636	1.38 (0.98–1.96)	
>636	1.94 (0.64–1.37)	

*IRR, incidence rate ratio. †Indicates global p value.

In addition, we investigated the temporal correlation between the epidemics and the litchi harvests to test whether AME cases occurred earlier in areas that harvested litchis earlier in the year. A Mann-Whitney U test was used to compare the distribution of the week numbers (1–52) of cases in the 5 districts that harvested litchis during May–June (group 1) to the distribution of the week numbers of cases in the 5 districts that harvested litchis during June–July (group 2). The test was performed in R (version 2.12.1; R Development Core Team, R Foundation for Statistical Computing, Vienna, Austria).

### Ethical Considerations

This study was approved by the Clinical Research Committee of Institut Pasteur, and ethical clearance was obtained from the NIHE Institutional Review Board. Informed consent for participation in the study was obtained from each child’s legal representative (parent, guardian, or other legally recognized person as defined by Vietnamese national laws).

## Results

### Spatial and Temporal Distribution of Cases

**Figure 2:**
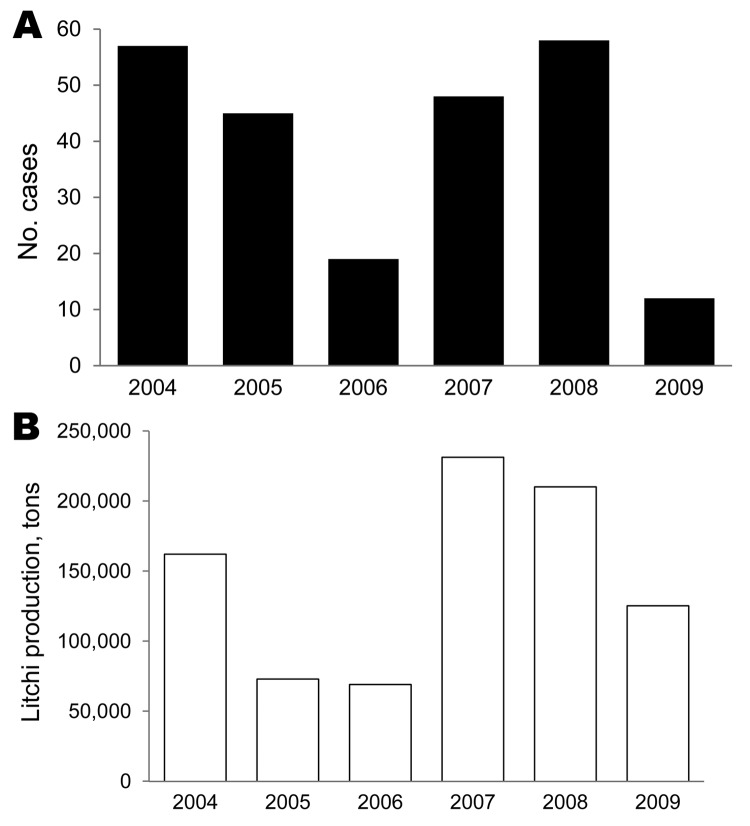
Temporal evolution of Ac Mong encephalitis (AME) and litchi cultivation, Bac Giang Province, Vietnam, 2004–2009. A) Annual number of AME cases; B) annual litchi production.

**Figure 3:**
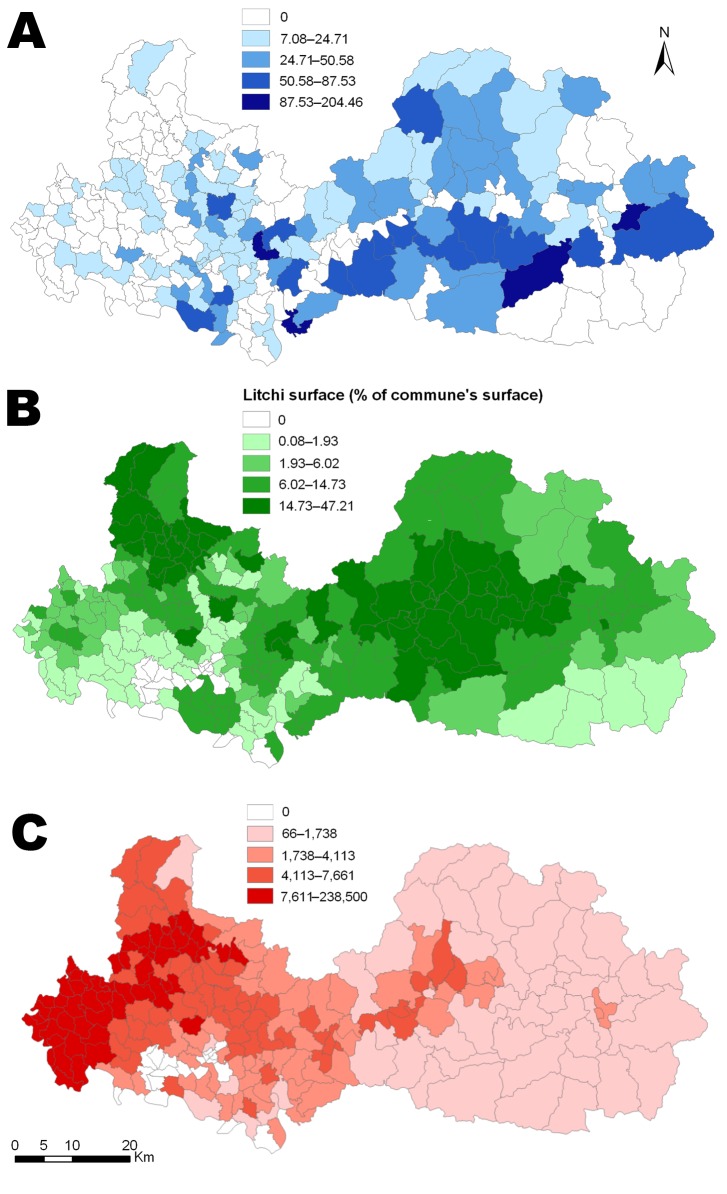
Commune-level maps of Bac Giang Province, Vietnam, 2004–2009. A) Cumulative incidence rate per 100,000 inhabitants of Ac Mong encephalitis. B) Mean percentage of commune surface area devoted to litchi cultivation. C) Mean poultry density (no. per km^2^).

During the study period, 239 children met the inclusion criteria. Their median age was 5 years (interquartile range 2–7.5), and the male:female ratio was 1.21:1. Temporally, the number of AME cases in the study decreased from 57 in 2004 to 19 in 2006, then increased to 58 in 2008 and fell to 12 in 2009 (Figure 2, panel A). The annual litchi production in the province is plotted for comparison purposes (Figure 2, panel B). Spatially, we estimated the cumulative incidence of the disease at the commune level during the study period, which ranged from 0 to 205 cases per 100,000 inhabitants (Figure 3, panel A). The highest incidences were mainly found in communes in Luc Nam, Luc Ngan, and Son Dong districts, accounting for 56% of all cases. The annual number of cases per commune ranged from 0 to 6. For comparative analysis, we determined the mean proportion of the communes’ surface devoted to litchi cultivation over the study period (Figure 3, panel B).

### Clinical and Laboratory Results

Detailed clinical and biologic results were available for 88 patients (Table 2). This subgroup of patients did not significantly differ from the whole group for all common measured characteristics. Children were hospitalized within 1 day (median) after symptom onset; most had fever (89%), seizures (82%), and vomiting (64%). At admission, meningeal signs (85%), altered mental status (87%), or coma (10%) were commonly seen. During hospitalization, limb paralysis developed in 13% of children. Twenty-two (25%) of the 88 children died of the disease. Spinal tap was performed in 75 children; CSF was transparent for 73 (97%) children. Leukocyte count in CSF, available for 16 children, was slightly elevated (median 20 cells/mm^3^ [reference <5 cells/mm^3^]), and with lymphocyte predominance (median 57%). Cranial computed tomography scans were performed in 50 children; no change was detected in 58% of children; edema in 28%; and focal signs in 14%.

**Table 2:** Clinical and laboratory features of Ac Mong encephalitis patients, Bac Giang Province, Vietnam, 2004–2009*

Feature	No. (%)	Median (IQR)
Sex, n = 88		
F	41 (47)	NA
M	47 (53)	NA
Age, y, n = 88		
<2	16 (18)	NA
2–4	27 (31)	NA
4–6	19 (22)	NA
6–15	26 (30)	NA
Symptoms/signs before and at admission		
Headache, n = 62	36 (58)	NA
Fever,† n = 85	76 (89)	NA
Temperature at admission, °C, n = 87	NA	38.2 (38.0–39.0)
Seizures, n = 87	71 (82)	NA
Altered mental status, n = 75	65 (87)	NA
Coma, n = 70	7 (10)	NA
Meningeal symptoms,‡ n = 67	57 (85)	NA
Limb paralysis, n = 75	4 (5)	NA
Cranial nerve palsy, n = 60	2 (3)	NA
Vomiting, n = 58	37 (64)	NA
Diarrhea, n = 52	4 (8)	NA
Days between onset and admission, n = 88	NA	1 (0–2)
Blood analysis	NA	NA
Leukocytes, × 10^9^ cells/L, n = 85	NA	17.5 (10.7–22.0)
Lymphocytes, %, n = 72	NA	19 (13–29)
Platelets, × 10^9^/L, n = 68	NA	254.5 (207.5–309.5)
Hemoglobin, g/L, n = 70	NA	114 (100–130)
Glucose, mmol/L, n = 72	NA	5.0 (3.2–6.1)
CSF sample		
Leukocytes, × 10^9^ cells/L, n = 16	NA	20 (12.5–85)
Lymphocytes, %, n = 18	NA	57 (40–75)
Protein level >0.5 g/L, n = 66	9 (14)	NA
Transparent appearance of CSF, n = 75	73 (97)	NA
Increased CSF pressure, i.e., >20 cm H_2_0, n = 70	35 (50)	NA
Cranial CT scan, n = 50		
Normal	29 (58)	NA
Diffuse edema	14 (28)	NA
Evidence of brain herniation	4 (8)	NA
Hemorrhagic stigma	3 (6)	NA
Intraparenchymal hypodensity	4 (8)	NA
Cortico-subcortical atrophy	8 (16)	NA
Liver enzymes, n = 17		
ALT, U/L	NA	42 (37–58)
AST, U/L	NA	58 (43–76)

*IQR, interquartile range; NA, not applicable; CSF, cerebrospinal fluid; CT, computed tomographic; ALT, alanine aminotransferase; AST, aspartate aminotransferase. †Observed by parents before admission or measured at admission (>38°C). ‡Either meningitis signs, stiff neck, Kernig’s sign, meningitis rash.

Results of Multiple Analyte Profiling are shown for selected analytes in the Technical Appendix Figure. interferon-α_2_ levels (mean ± SEM) were elevated in the serum of patients who survived AME (538.5 ± 196.6) compared with their sibling controls (56.15 ± 55.09) (p = 0.17). Interleukin-8 (IL-8) levels were elevated in the CSF of AME patients of both outcomes but were higher in those who died (930.3 ± 328.7) than in those who survived (237.6 ± 118.4) (p = 0.095). Serum IL-6 also was detected in patients of both outcomes, with higher levels in patients who died (132.2 ± 68.5) than in sibling controls (0.875 ± 0.425) (p = 0.029). IL-6 was elevated in the CSF of patients who died (430.1 ± 148.5) and in those who survived the disease (2151.8 ± 1934.6).

### Ecologic Regression Results

The final multivariate model resulted in 3 factors independently associated with AME (Table 3). A positive association between disease incidence and litchi surface proportion was found: the IRRs were 1.52 (95% CI 0.90–2.57), 2.94 (95% CI 1.88–4.60), and 2.76 (95% CI 1.76–4.32) for second, third, and fourth quartiles, respectively, compared with the lowest quartile. A reduced risk was associated with density of poultry: the IRRs were 0.62 (95% CI 0.43–0.91), 0.61 (95% CI 0.42–0.89), and 0.25 (0.15–0.43) for second, third, and fourth quartiles, respectively, compared with the lowest quartile. Relative humidity was negatively associated with disease incidence: the IRRs were 0.84 (95% CI 0.61–1.16) and 0.35 (95% CI 0.23–0.54) for second and third terciles, respectively, compared with the lowest tercile.

**Table 3:** Multivariate analysis of Ac Mong encephalitis, Bac Giang Province, Vietnam, 2004–2009

Variable/category	IRR* (95% CI)	p value†
Litchi surface, % commune surface		
<1.49	1	<0.001
1.49–5.84	1.52 (0.90–2.57)	
5.84–12.92	2.94 (1.88–4.60)	
>12.92	2.76 (1.76–4.32)	
Poultry density, no./km^2^		
<1,449.79	1	<0.001
1,449.79–3,715.18	0.62 (0.43–0.91)	
3,715.18–7,065.25	0.61 (0.42–0.89)	
>7,065.25	0.25 (0.15–0.43)	
Mean humidity, %, January–April		
<82.07	1	<0.001
82.07–83.55	0.84 (0.61–1.16)	
>83.55	0.35 (0.23–0.54)	

*IRR, incidence rate ratio. †Indicates global p value.

### Temporal Correlation between the Epidemics and the Litchi Harvests

**Figure 4:**
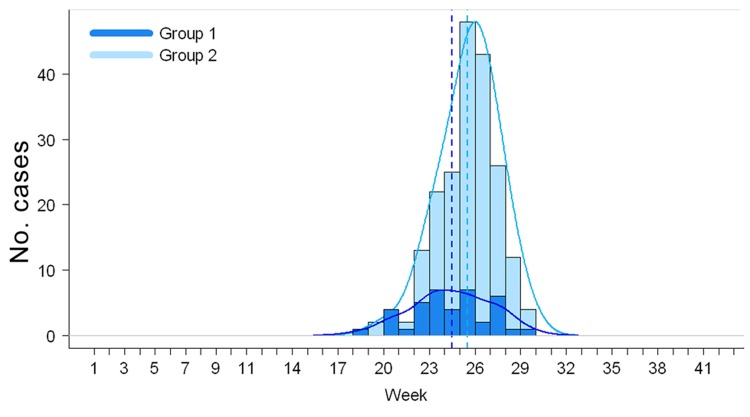
Distribution of weekly numbers of Ac Mong encephalitis cases, Bac Giang Province, Vietnam, 2004–2009, by 2 groups of districts: districts that harvested litchi during May–June (group 1) and districts that harvested litchi during June–July (group 2). Kernel densities are shown in solid lines and medians in dotted lines.

Figure 4 displays the weekly distributions of AME cases and associated kernel densities for the districts that harvested litchis during May–June (group 1) and during June–July (group 2). The Mann-Whitney U test showed that the distributions of the week numbers differed significantly (p = 0.007) between the 2 groups, with a delay of 1 week for group 2 compared with group 1.

## Discussion

We found evidence for a spatiotemporal association between the outbreaks of unknown encephalitis in Bac Giang Province and litchi cultivation. The ecologic regression analysis demonstrated that the annual risk for AME in a commune increased with the proportion of litchi-cultivated surface and that the epidemics occurred earlier in the districts that harvested litchis during May–June than in those that harvested litchis during June–July.

Similar to the clinical features of Chandipura encephalitis (*3*), i.e., acute encephalitis with rapid onset, those reported here suggest a viral etiology. Unfortunately, all viral investigations have thus far been uninformative. New techniques, such as high-throughput sequencing and resequencing micro-arrays, are currently performed on serum, CSF, culture supernatants, and brain homogenates from suckling mice inoculated intracerebrally with patients’ CSF. Although electron microscopy of brain tissue is a standard method of identifying new viral pathogens in encephalitis syndromes, we were unable to obtain brain tissue specimens through autopsy because of cultural barriers.

The association between litchis and acute encephalitis remains unclear. As with other emerging viruses, we face a multifactorial problem that seems to have litchi fruit production and harvest as its focal point. One possible scenario is that fruit-bearing litchi trees can attract bats, which might be the reservoir for the putative pathogen. Mosquitoes could feed on the infected bats and transmit the virus to humans who have insufficient protection against mosquito bites. Several species of bats were identified in the province, such as the frugivorous bats *Rousettus leschenaultii*, which can feed on litchi. These bats’ highly gregarious, cave dwelling, and migratory characteristics facilitate their role in virus carrying and circulation (*10*).The bat population density is high during April–September (which includes the time of litchi harvest) before migration during October–March.

In addition, several mosquito species were identified in Bac Giang Province: *Anopheles vagus*, *Armigeres subalbatus*, *Culex tritaeniorhynchus*, *Cx. vishnui*, *Cx. gelidus*, *Cx. fuscocephalus*, *Cx. quinquefasciatus*, and *Cx. bitaeniorhynchus*. Although the peak incidence of May–July correlates with the rice paddy breeding and development of *Culex* spp. mosquitoes, the paddy fields area was negatively associated with the risk for disease in univariate analysis. No data were collected on vector densities in Bac Giang.

Other modes of transmission, e.g., direct contact with litchis contaminated by bat saliva, urine, or guano (*11*) or with other vectors, such as insects found in litchi trees or phlebotomine sand flies, as in the case of Chandipura virus (*12*), cannot be excluded. Deforestation in Bac Giang to develop the growing of litchi trees because of their high economic value also might have disrupted the ecologic equilibrium of the province, leading to the emergence of a new vector-borne disease (*13*). The putative virus also might be exclusively human; use of human feces as fertilizers to enhance litchi growth in these plantations might have contaminated the soil with enteroviruses, which are known to cause fatal encephalitis in deprived children (*14*).

Last, a toxic origin might be possible; in India, a toxic weed, *Cassia occidentalis*, caused an acute hepatomyoencephalopathy syndrome, which was first assumed to be viral encephalitis (*15*). However, in our study, the presence of fever and meningeal symptoms and the absence of high elevation of liver enzyme favor a viral etiology rather than a toxic origin. Additionally, we demonstrated that children with AME harbor elevated levels of type I interferon in serum and interferon-inducible cytokines, such as IL-6 and IL-8 in CSF, compatible with an infectious process (*16*,*17*); however, these cytokines are expected to be elevated in any inflammatory process.

The protective effects of poultry density and mean relative humidity during January–April (pre–epidemic period) are more difficult to interpret. With respect to the hypothesis of an arbovirus-mediated pathogenesis, a possibility is that poultry is a preferred host for the putative vector (*18*). Regarding negative correlation with humidity, above a certain limit, high relative humidity can be harmful to insects; insects or their eggs can drown or be infected more readily by pathogens (*19*). Litchi growing also depends on climate; ideal conditions include a brief dry, cool, and frost-free winter to lead to flowering, followed by warmer temperatures and moderate rainfall and humidity during fruit development and harvest (*20*). Still, climatic variations alone could not explain the spatial differences in disease risk between communes of the same province. Moreover, even if the association is not proved to be causal, the persistence of both variables (litchi surface and mean humidity) in the final model suggests that both factors are independently correlated with the spatiotemporal patterns of the disease. Definitive identification of the infectious agent will help clarify these factors associated with AME incidence.

Because our study concerns an ecologic investigation, the relationship between litchis and AME cannot be inferred at an individual level. Another caveat of the study concerns the nonspecific case definition based on clinical features and negative JEV serologic test results. False-negative results have been observed for JEV serologic tests performed soon after onset of symptoms (*21*); nevertheless, the widespread use of JE vaccination, introduced in 1997, has considerably reduced JE incidence in the region. In addition, after JE vaccine introduction in the WHO Expanded Program on Immunization, AES surveillance might have been intensified, and an apparent increased incidence of AES might be simply an artifact of more active surveillance.

Artifact is not likely to account for the cases described in this manuscript, however, because they were identified clinically among patients seeking care at the hospital with AES rather than through public health surveillance. Conversely, surveillance might have failed to capture some cases because of the rapid deaths of infected children (e.g., those who died before reaching the hospital).

Apart from these cases, given the severity of the disease, we can assume that all parents, including those who lived in remote villages, sought medical attention. Unfortunately, because of logistical difficulties, systematic data and sample collection and analysis could not be conducted for all case-patients in Bac Giang hospital. Nevertheless, we have no reason to believe that cases for which biologic data were available (Table 2) differed from other cases.

Our ecologic analysis of outbreaks of acute encephalitis of unknown origin during the litchi harvest period in Bac Giang Province strengthens the hypothesis that litchis might play a role in these outbreaks by showing that litchi cultivation was spatially and temporally associated with AME. This finding can be useful to guide future prospective studies. The suggested role of litchi trees needs to be more thoroughly investigated to explain disease ecology. Further research should include investigating the specific agricultural practices linked to litchi cultivation, distribution of tasks among adults and children, locations where these activities are conducted, and other activities undertaken around litchi fields before and during the epidemic period; conducting entomologic surveys around litchi plantations; and analyzing potential reservoirs and hosts. Last, research efforts should be continued to identify the causative agent.

Technical Appendix Cerebrospinal fluid and serum concentrations of interferon-α2, interleukin (IL) 8, and IL-6 in 10 children with Ac Mong encephalitis (5 who survived and 5 who died of the disease) and 4 asymptomatic siblings, Bac Giang Province, Vietnam, 2004–2009.
