# Supplementary material for: Litchi–associated Acute Encephalitis in Children, Northern Vietnam, 2004–2009
Source: Emerg Infect Dis. 2012 Nov;18(11):1817–24. doi: 10.3201/eid1811.111761 (PMC3559149; doi:10.3201/eid1811.111761)
Supplement: Technical Appendix — Cerebrospinal fluid and serum concentrations of interferon-α2, interleukin (IL) 8, and IL-6 in 10 children with Ac Mong encephalitis (5 who survived and 5 who died of the disease) and 4 asymptomatic siblings, Bac Giang Province, Vietnam, 2004–2009. [file 11-1761-Techapp-s1.pdf]

Article DOI: http://dx.doi.org/10.3201/eid1811.111761

Litchi–associated Acute Encephalitis in Children, Northern Vietnam, 2004–2009

Technical Appendix

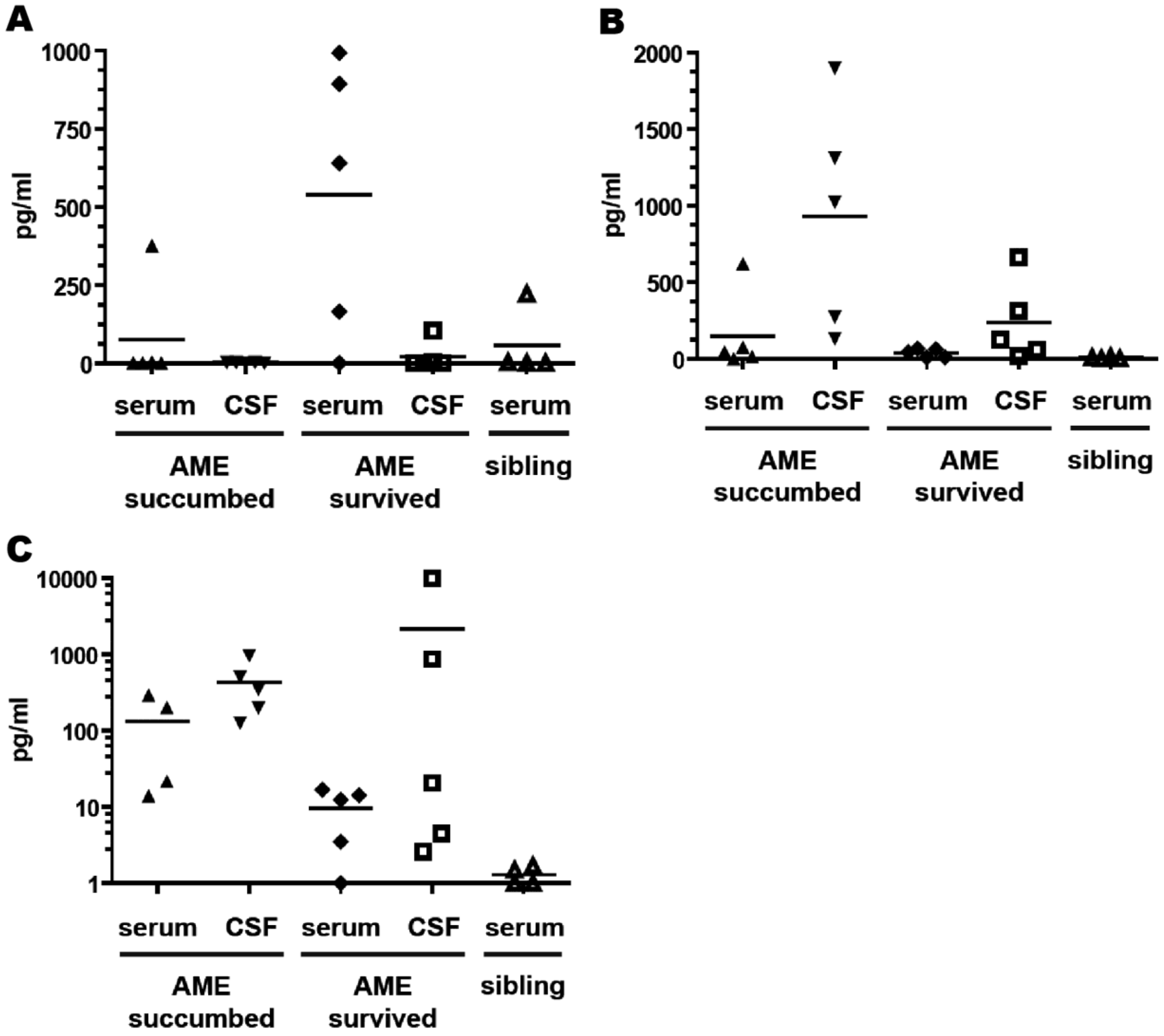

Technical Appendix Figure. Cerebrospinal fluid and serum concentrations of 3 immune mediators: interferon-α2 (IFN-α_2) (A]), interleukin 8 (IL-8) (B), and IL-6 (C) in 10 children with Ac Mong encephalitis (5 who survived and 5 who died of the disease) and 4 asymptomatic siblings as controls, Bac Giang Province, Vietnam, 2004–2009.
